# Practical Enhancements in Current Density and Power Generation of Bifacial Semitransparent Ultrathin CIGSe Solar Cells via Utilization of Wide Bandgap Zn‐Based Buffer

**DOI:** 10.1002/advs.202105436

**Published:** 2022-02-23

**Authors:** Dongryeol Kim, Sang Su Shin, Yonghee Jo, Sang Min Lee, Seung Kyu Ahn, Jun‐Sik Cho, Jae Ho Yun, Ho Seong Lee, Joo Hyung Park

**Affiliations:** ^1^ Photovoltaics Research Department Korea Institute of Energy Research (KIER) Daejeon 34129 Republic of Korea; ^2^ School of Materials Science and Engineering Kyungpook National University Daegu 41556 Republic of Korea; ^3^ Department of Electrical Engineering and Smart Grid Research Center Jeonbuk National University Jeonju 54896 Republic of Korea

**Keywords:** albedo effect, atomic layer deposition, bifacial compatible efficiencies, semitransparent ultrathin Cu(In_1‐x_,Ga_x_)Se_2_, solar cells, Zn(O,S) buffers

## Abstract

Among many building‐integrated semitransparent photovoltaics (BISTPVs), semitransparent ultrathin (STUT) Cu(In_x_,Ga_1‐x_)Se_2_ (CIGSe) solar cells are distinguishable due to their potential high power conversion efficiency (PCE) among other thin‐film solar cells, versatile applicability based on thin film deposition processes, high stability consisting of all inorganic compositions, and practical expandability to bifacial applications. However, the fundamental trade‐off relationship between PCE and transparency limits the performance of BISTPV because implementing a higher semitransparency lowers the optical budget of incoming light. To expand the available optical budget and to enhance the PCE while maintaining a suitable transparency in STUT CIGSe solar cell with single‐stage coevaporated 500‐nm‐thick absorber, an atomic layer deposited wide bandgap Zn(O,S) buffer is introduced as the replacement of conventional CdS buffer, which partially limits incoming light less than 520 nm in wavelength. As a replacement result, more incoming light becomes valid for power conversion, and the short circuit current density (*J*_sc_) has increased comparatively by 17%, which has directly lead to a large increase in PCE up to 12.41%. Furthermore, Zn(O,S) buffer in the STUT CIGSe solar cell also has enhanced the bifacial compatible efficiency (BCE), which has increased to 14.44% at 1.3 sun and 19.42% at 2.0 sun.

## Graphical abstract

Performance of semitransparent ultrathin (STUT) Cu(In,Ga)Se_2_ (CIGSe) solar cell is enhanced by expanding the optical budget with wide bandgap Zn‐based buffer and considering the albedo effect. The best PCE of STUT CIGSe solar cell is 12.41%, and the best bifacial compatible efficiency linearly increases as increasing total illumination, which reach 14.44% at 1.3 sun and 19.42% at 2.0 sun.

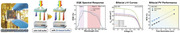

## 1 Introduction

Building‐integrated photovoltaics (BIPVs) and building‐integrated semitransparent photovoltaics (BISTPVs) as eco‐friendly and renewable energy sources are an ongoing research subject not only for green energy generation^[^ ^1^, ^2^, ^3^ ^]^ but also for architectural components.^[^ ^4^, ^5^ ^]^ As one of the building elements, semitransparent thin‐film solar cells are practically and diversely applicable to building exteriors as an energy harvester along with aesthetic embellishment by providing semitransparency. In particular, semitransparent ultrathin (STUT) Cu(In_1‐x_,Ga_x_)Se_2_ (CIGSe) thin‐film solar cells have several advantages. First, a STUT CIGSe solar cell with an ultrathin absorber layer (<500 nm) can not only reduce the process cost by frugal usage of elemental sources but also alleviate the installation by saving costs for frames and construction sites, considering that its application approaches to windows and building envelopes can be an attachment rather than an installation. In addition, the power conversion efficiency (PCE) is reported to be stable upon exposure to an external environment.^[^ ^6^, ^7^, ^8^ ^]^ Finally, the STUT CIGSe solar cell can also be utilized as a bifacial solar cell without severe modification because it can also absorb light from the rear side.^[^ ^9^, ^10^, ^11^ ^]^

Although the PCE of CIGSe solar cells with an absorber layer thickness of ≈2 µm utilizing a CdS buffer layer has reached 22.6%,^[^ ^12^ ^]^ the need to replace CdS with a nontoxic wide bandgap material is still continuing. For this reason, a Zn‐based buffer layer has been studied to replace a conventional CdS buffer,^[^ ^13^, ^14^, ^15^ ^]^ and the best PCE recently achieved 23.35% using Zn‐based Cd‐free buffer in a Cu(In_x_,Ga_1‐x_)(S_y_Se_1‐y_)_2_ (CIGSSe) solar cell structure,^[^ ^16^ ^]^ which is a comparable result to the PCE with CdS buffer. Meanwhile, the highest reported PCE from CIGSSe solar cell with Zn(O,S) buffer using atomic layer deposition (ALD) was comparatively low, which is 19.78%.^[^ ^17^ ^]^ In addition, that of CIGSe solar cell with Zn(O,S) buffer using ALD is even lower, which is 18.3%.^[^ ^18^ ^]^ In the case of STUT CIGSe solar cells, the best PCE of 10.54% without antireflection coating (ARC) was reported using a single‐stage coevaporation process previously.^[^ ^19^ ^]^ In addition, using three‐stage coevaporation, the best PCE of 12.9% was recently reported by Li et al.^[^ ^20^ ^]^ In general, the semitransparent application has a trade‐off relationship between PCE and semitransparency due to a limited optical budget of incoming light, and the importance of wide bandgap buffer is more highlighted in STUT CIGSe solar cell because the adoption of wider bandgap buffer may provide chances to expand the total optical budget without increasing of processes and complicating modification of structure as well as to substitute a toxic material with an eco‐friendly one. Hence, the study of STUT CIGSe solar cells with ALD‐grown wide bandgap Zn(O,S) buffer can suggest beneficial characteristics of thin‐film solar cells with a wider bandgap buffer, broaden deeper understanding of the optical budget, and finally contribute to the power generation enhancement of bifacial and/or semitransparent solar cells.

To make the most use of the limited optical budget of incoming light by adopting ALD‐grown wide‐bandgap Zn(O,S) buffer in a STUT CIGSe solar cell, three different experiments were conducted. These subjects include (i) CIGSe surface passivation by wide‐bandgap ZnS to suppress surface recombination and increase open‐circuit voltage (*V*_oc_), (ii) optimization of Zn(O,S) to find an adequate work function with a sufficiently large bandgap and increase short‐circuit current density (*J*_sc_), and (iii) studies on future possibilities by revealing the wide‐bandgap buffer effects on bifacial solar cell applications.

To begin with CIGSe surface passivation for the first, reducing surface recombination by inserting a passivation layer between the buffer and absorber has been researched consistently in various solar cells, such as Si, CIGSe, Cu_2_ZnSn(S,Se)_4_ (CZTSSe), and perovskite solar cells^[^ ^21^, ^22^, ^23^, ^24^ ^]^ using various materials, including Al_2_O_3_,^[^ ^23^ ^]^ SiO_2_,^[^ ^25^ ^]^ and ZnS.^[^ ^26^, ^27^, ^28^, ^29^ ^]^ In these studies, a decrease in surface recombination,^[^ ^24^ ^]^ an increase in the external quantum efficiency (EQE) spectrum in the longer wavelength region,^[^ ^23^ ^]^ and an increase in the current density (*J*_sc_) of ≈4%^[^ ^25^ ^]^ were reported to be beneficial for surface passivation. In other reports, increases in the open‐circuit voltage (*V*_oc_) and fill factor (FF) were also addressed,^[^ ^26^, ^27^, ^28^, ^29^ ^]^ depending on the absorber and device structure properties.

The second experiment is to optimize the ALD‐grown Zn(O,S) buffer in the STUT CIGSe solar cell structure. ZnO_1−x_S_x_ has a variable wide‐bandgap that can be controlled by changing *x* between 0.0 and 1.0 (i.e., Zn(O,S) bandgap of 2.8 to 3.7 eV),^[^ ^30^ ^]^ while CdS has a relatively small fixed bandgap (2.4 eV), which may partially limit incoming light under 520 nm depending on the thickness.^[^ ^31^ ^]^ Accordingly, the work function of Zn(O,S) also varies by changing the ratio of oxygen and sulfur contents. Hence, the optimal conduction band offset (CBO) at the Zn(O,S) buffer/CIGSe absorber interface can be found by adjusting the O:S ratio^[^ ^32^ ^]^ for a fixed bandgap of the CIGSe absorber. In this report, the bandgap of the CIGSe absorber was fixed at 1.18 eV. Researchers report that the conduction band alignment of the absorber and buffer may form a spike (positive CBO) or cliff (negative CBO),^[^ ^33^, ^34^ ^]^ which can be calculated by subtracting the conduction band maximum (CBM) of the absorber from that of the buffer. It has also been reported that the photogenerated current can be blocked if the spike is too high and that *V*_oc_ can abruptly decrease if the cliff is formed. To ensure barrier‐free migration of photocarriers at the interface without the performance degradation of the CIGSe solar cell, a spikelike band alignment of ≈0.3 eV is required for the CIGSe absorber bandgap of 1.15 eV.^[^ ^29^ ^]^ Therefore, as addressed by other researchers,^[^ ^13^, ^17^, ^35^ ^]^ the adoption of Zn(O,S) as a substitution for CdS can exclude toxic elements and facilitate the utilization of the optical budget by widening the wavelength range of incoming light into the CIGSe absorber.

**Figure 1:**
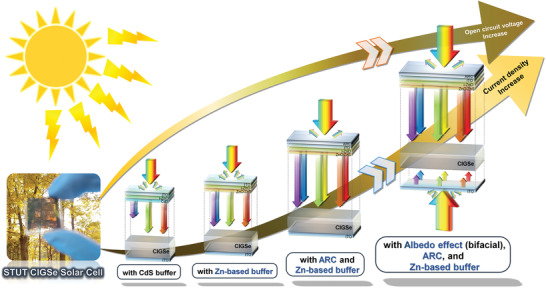
Schematic of STUT CIGSe solar cell structures as increasing the optical budget.

Finally, the third experiment is an extreme utilization of a STUT CIGSe solar cell for bifacial applications with total illumination up to 2 sun. The bifacial application of STUT CIGSe solar cells may have several advantages considering that it allows the utilization of direct and reflected sunlight from both the front and rear sides of solar cells and thereby increases charge carrier generation,^[^ ^36^ ^]^ which means an enlarged optical budget from the point of solar cell power generation. In particular, the bifacial STUT CIGSe solar cell has the following favorable features. (i) The direct bifacial application is possible without any special modification from the monofacial STUT CIGSe solar cell structure. (ii) The bifacial STUT CIGSe solar cell may compensate for the relatively low *J*_sc,_ which is an underlying general property of monofacial STUT CIGSe. As a consequence of *J*_sc_ compensation, solar cell power generation increases, and the economic feasibility can be enhanced. (iii) The adoption of Zn(O,S) buffer into the STUT CIGSe solar cell may further flourish the optical budget of incoming light from the front side because the STUT CIGSe solar cell efficiently absorbs light from the relatively shorter wavelength region rather than the longer wavelength region owing to the limited thickness. However, reminding that PCE is defined as a measure of solar cell performance, which is the ratio of output power to input power when the input power is 1 sun considering monofacial solar cells, the performance of bifacial solar cells needs a new definition of efficiency, which can encompass additional power generation by the albedo effect from the rear side in practical fields. In other words, the total input power into a bifacial solar cell is different according to the additional rear‐side illumination by the albedo effect, although front‐side illumination is fixed at 1 sun. Consequently, the additional input from the rear side may increase the total illumination by more than 1 sun (i.e., between 1 and 2 sun, typically 1.2–1.3 sun assuming 0.2–0.3 sun albedo effect in practical fields)^[^ ^37^, ^38^ ^]^ and contribute to generating more power in STUT CIGSe solar cells despite the same PCE. In this regard, several researchers defined new terms, such as “apparent efficiency,” “(bifacial) equivalent efficiency,” and “gain‐efficiency product.”^[^ ^11^, ^39^, ^40^ ^]^ These terms of efficiency represent the bifacial solar cell's compatible efficiency (i.e., power generation, to be more exact) to monofacial solar cells, which have equivalent power generation performance per unit area under the same conditions except for additional rear‐side illumination.^[^ ^41^, ^42^ ^]^ In this report, bifacial compatible efficiency (BCE) was defined as the term for bifacial power generation ratio per unit area at a total input power ranging from 1.0 to 2.0 sun (i.e., The front‐side illumination is fixed at 1.0 sun, and BCE at 1.0 sun is the same power generation as PCE), which represents the bifacial solar cell's compatible efficiency to monofacial solar cells, including the consideration of the albedo effect. Hence, BCE encompasses not only the power generation based on PCE but also the practically enhanced amount owing to the albedo effect (i.e., precisely, BCE represents the total power generation (rather than the power conversion efficiency) under 1.0 to 2.0 sun with double‐side illumination (1 sun from the front‐side illumination and 0 to 1 sun from the rear‐side). In comparison, PCE represents the power conversion efficiency or power generation under 1 sun front‐side illumination only. The overall schematic of those three above mentioned experiments were digested in **Figure** **1**.

This study focused on utilizing and enlarging the optical budget of STUT CIGSe solar cells, which included the adoption of a ZnS passivation layer, the optimization of Zn(O,S) buffer, the comparison of photovoltaic (PV) properties in Zn(O,S)/CIGSe solar cells with those in conventional CdS/CIGSe solar cells, and the bifacial application for higher PV performance and economic feasibility. In addition, a dedicated zig for bifacial measurement was designed and prepared to measure the bifacial PV properties and eliminate any bias depending on the optical path of illumination. The performance of bifacial STUT CIGSe solar cells was measured using total illumination from 1 to 2 sun using double‐side illumination (while maintaining 1 sun illumination on the front side), supposing an environmental albedo effect in an actual field and including an extreme case. Therefore, these findings are helpful to understand the optical budget of STUT CIGSe solar cells, maximize the utilization of incoming light, and further increase the PCE and BCE of semitransparent solar cells.

## 2 Results and Discussion

### 2.1 Engineering of the ZnS Passivation Layer, Zn(O,S) Buffer Layer, and Band Structure

To make the maximal use of the limited optical budget of incoming light, the first step starts with the surface passivation of the CIGSe absorber for effective utilization of photogenerated carriers without a severe loss. A ZnS passivation layer was introduced to resolve the surface or interface defect‐related performance deficiencies in the STUT CIGSe solar cell structure. Considering that the STUT CIGSe absorber is Cu poor, which is prone to form Cu and Se vacancies (*V*_Cu_ and *V*_Se_), the possibility of their presence at the interface is also likely including other defects in CIGSe.^[^ ^43^, ^44^, ^45^ ^]^ In addition, Zn(O,S) also have several defect related uncertainty issues.^[^ ^46^, ^47^, ^48^ ^]^ Moreover, defects at the buffer/CIGSe interface in the form of sodium compounds and oxides can be formed during the NaF post‐deposition treatment (PDT) and air exposure prior to buffer deposition^[^ ^49^ ^]^ and may increase carrier recombination by trapping charges. In the conventional CIGSe solar cell process, the wet chemical bath deposition (CBD) process for CdS buffer is known to clean the surface by removing impurities such as Na_2_CO_3_, In_2_O_3_, and Ga_2_O_3_,^[^ ^50^ ^]^ while the ALD process for Zn(O,S) buffer cannot effectively provide etching. There is a need to employ a ZnS passivation layer, which can eliminate the effect of trapped charges at the defect sites of the Zn(O,S)/CIGSe interface by isolating them. More discussions can be found in our previous report.^[^ ^29^ ^]^

**Figure 2:**
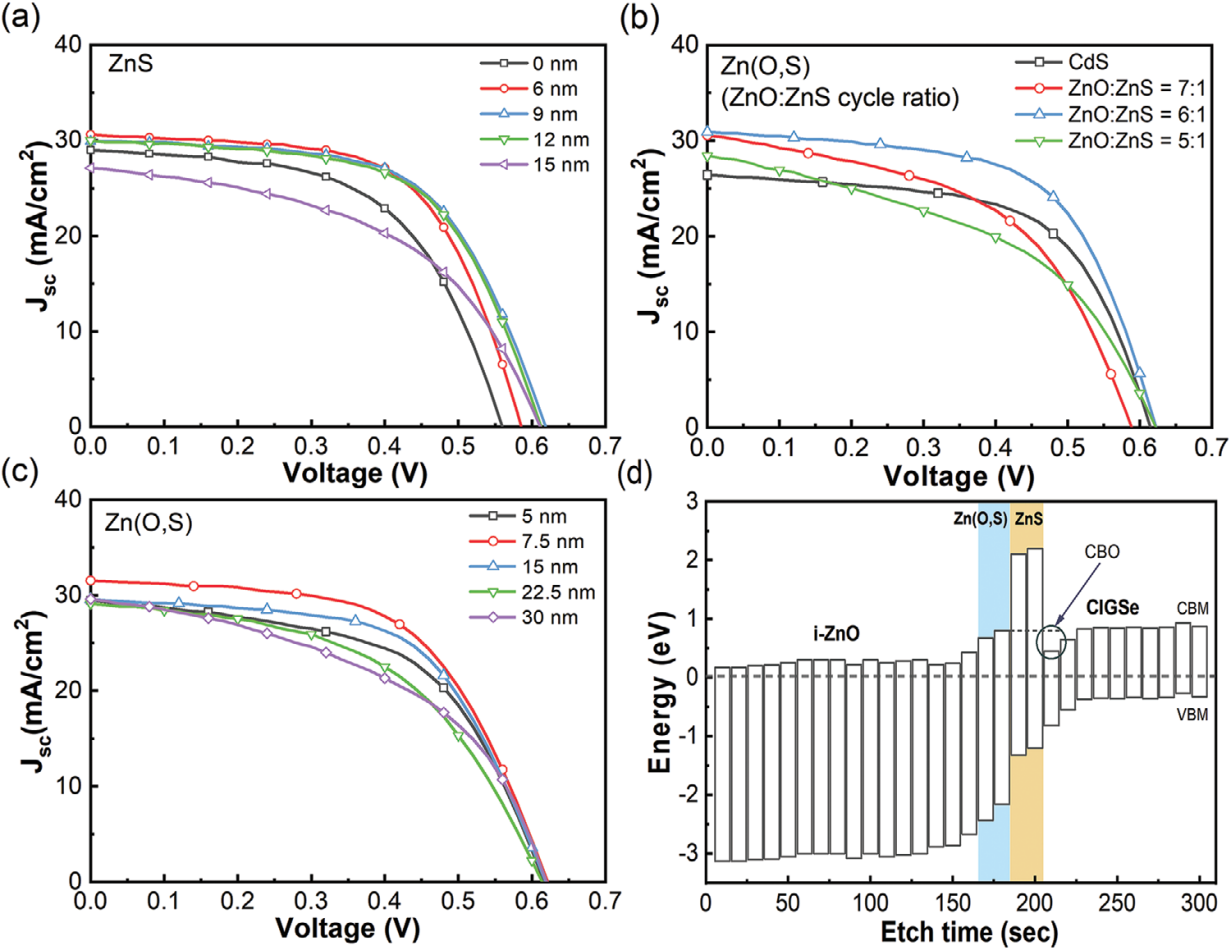
*J*–*V* curves of STUT CIGSe solar cells with a) various ZnS passivation thicknesses, b) various ZnO:ZnS cycle ratios of Zn(O,S) buffer, c) various Zn(O,S) buffer thicknesses, and d) the reconstructed band structure based on measured results from XPS analyses and material bandgaps.

By controlling the thickness of ZnS, we investigated the change in PV parameters of STUT CIGSe solar cells. **Figure** **2**a and **Table** **1**a show the *J*–*V* curves of STUT CIGSe solar cells with different thicknesses of ZnS layers. The thickness of the ZnS thickness for passivation was varied from 0 to 15 nm, while that of the Zn(O,S) buffer layer was fixed at 7.5 nm. The best PCE of the STUT CIGSe solar cell with a 9‐nm‐thick ZnS passivation layer was 11.18%. Increasing the ZnS thickness from 0 to 9 nm caused an increase in *V*_oc_, which saturated the *V*_oc_ value to 0.6185 eV by passivating interface defects. The series resistance (*R*_s_) increased from 1.112 to 2.534 Ω cm^2^, and the shunt resistance (*R*_sh_) also increased from 331.7 to 1299 Ω cm^2^, where *R*_s_ is related to the electrical property and connection of each layer, and *R*_sh_ is relevant to the overall leakage current in a device and the photogenerated current loss by recombination.^[^ ^27^ ^]^ Hence, as the thickness of the ZnS passivation layer increases to 9 nm, the increased *R*_s_ reflects the relatively high resistivity of ZnS, and the increased *R*_sh_ means that the leakage or loss of current is decreased due to the interface passivation effect. The diode leakage current density (*J*_0_) and ideality factor (*A*) also vary depending on the thickness of the ZnS layer. When the thickness of the ZnS passivation layer reached 9 nm, *J*_0_ decreased to 2.346 × 10^−5^ mA cm^−2^, which is more than two orders of magnitude smaller than that of STUT CIGSe without ZnS passivation (i.e., ZnS 0 nm in Figure 2 and Table 1). At the same time, the *A* value decreased to 1.191, which indicated that the diode inside the STUT CIGSe solar cell was close to an ideal diode with a severely controlled level of recombination mechanism. Hegedus and Shafarman^[^ ^51^ ^]^ reported that typical well‐behaving thin film solar cells have *A* value between 1.3 and 2.0, which indicates that major recombination occurs through the trap states in the space‐charge region of the absorber. In addition, they noted that an *A* value greater than 2 means that interface recombination is dominant. Hence, regarding the decreased *J*_0_ and *A* values, the STUT CIGSe solar cell with a 9‐nm‐thick ZnS passivation layer demonstrates the significantly well‐passivated surface of CIGSe with well‐suppressed recombination as well as competent diode qualities. Upon a further gradual increase in ZnS thickness from 9 to 15 nm, negative impacts gradually emerged, as confirmed by Figure S1 and Table S1 (Supporting Information). As the thickness of ZnS reached 15 nm, the *J*_sc_ and FF values decreased, while the saturated *V*_oc_ stayed in a similar range. The reason for these deteriorations can be attributed to the limited tunneling current through the ZnS passivation layer. The relatively thicker ZnS passivation may act as a barrier to photogenerated carriers in the CIGSe absorber, which disturbs the migration of carriers in the CIGS absorber from reaching the front and rear electrodes.

**Table 1:** Summary of PV and diode parameters of STUT CIGSe solar cells with various ZnS passivation thicknesses, various ZnO:ZnS cycle ratios of Zn(O,S) buffer, and various Zn(O,S) buffer thicknesses

(a) PV and diode parameters^a)^ of STUT CIGSe solar cells with various ZnS passivation thicknesses								
ZnS [nm]	*V*_oc_ [V]	*J*_sc_ [mA cm^−2^]	FF [%]	PCE [%]	*R*_s_ [Ω cm^2^]	*R*_sh_ [Ω cm^2^]	*A*	*J*_0_ [mA cm^−2^]
0 nm	0.5598	29.02	56.50	9.18	1.112	331.7	2.025	2.251 × 10^−3^
6 nm	0.5854	30.55	61.30	10.96	1.693	490.2	1.386	2.787 × 10^−4^
9 nm	0.6185	29.95	60.34	11.18	2.534	1299	1.191	2.346 × 10^−5^
12 nm	0.6123	29.98	60.08	11.03	2.314	925.9	1.573	5.474 × 10^−4^
15 nm	0.6112	27.07	49.43	8.18	2.172	216.6	2.034	2.679 × 10^−4^

(b) PV parameters of STUT CIGSe solar cells with various ZnO:ZnS cycle ratios of Zn(O,S) buffer				
Zn(O,S) (ZnO:ZnS cycle ratio of ALD)	*V*_oc_ [V]	J_sc_ [mA cm^−2^]	FF [%]	PCE [%]
CdS	0.6147	26.42	60.84	9.88
7:1	0.5887	30.55	50.53	9.09
6:1	0.6223	30.91	60.42	11.62
5:1	0.6221	28.39	45.85	8.10

(c) PV parameters of STUT CIGSe solar cells with various Zn(O,S) buffer thicknesses				
Zn(O,S) buffer thickness [nm]	*V*_oc_ [V]	*J*_sc_ [mA cm^−2^]	FF [%]	PCE [%]
5	0.6165	29.44	55.59	10.09
7.5	0.6212	31.55	58.11	11.39
15	0.6183	29.56	59.34	10.85
22.5	0.6137	29.15	50.42	9.02
30	0.6198	29.59	47.21	8.66

^a)^ The diode parameters including series resistance (*R*_s_), shunt resistance (*R*_sh_), ideality factor (*A*), and diode leakage current density (*J*_0_) are determined by the dark *J*–*V* curve.

After determining the optimum thickness of ZnS passivation, the ALD‐grown Zn(O,S) buffer layer was investigated by two different sets of experiments: one was to achieve the best CBO by adjusting the bandgap of Zn(O,S) buffer, and the other was to find an optimum thickness. During these two experiments, the thickness of ZnS passivation was fixed at 9 nm, which exhibited the highest *V*_oc_. In the first experimental set from the two different experiments, the composition of Zn(O,S) was varied to meet the ideal CBO value by controlling the ratios of ZnO to ZnS layer numbers in ALD cycles (i.e., ZnO:ZnS cycle ratio). The ZnO:ZnS cycle ratio ranged from 7:1 to 5:1 to increase the S content in Zn(O,S) buffer, which increased the Zn(O,S) bandgap and work function. Based on valence band maximum (VBM) values acquired from X‐ray photoelectron spectroscopy (XPS) analysis results and optical bandgap values calculated from optical measurement with Zn(O,S) buffers and an ultrathin CIGSe absorber, the optimal CBO close to 0.3 eV was expected at a ZnO:ZnS cycle ratio of 6:1. Comparing the *J*–*V* curves of STUT CIGSe solar cells with Zn(O,S) buffers of different ZnO:ZnS cycle ratios, the contribution of the optimal CBO was observed at the *V*_oc_ values. Figure 2b and Table 1b show that the *V*_oc_ values of STUT CIGSe solar cells with 5:1 and 6:1 Zn(O,S) buffers were even higher than those with CdS buffer, as confirmed by Figure S2 and Table S2 (Supporting Information). However, the *V*_oc_ of the CIGSe solar cell with 7:1 Zn(O,S) buffer was ≈33.6 mV lower than that with 6:1 Zn(O,S). These behaviors exactly matched the expected performance changes of CIGSe solar cells around the optimized CBO condition.^[^ ^33^ ^]^ In addition, as expected from the wider bandgaps of Zn(O,S), all STUT CIGSe solar cells with Zn(O,S) buffer exhibited higher *J*_sc_ values than those with CdS buffer owing to the increased optical budget. The reduction in *J*_sc_ in the STUT CIGSe solar cell with 5:1 Zn(O,S) buffer was attributed to the unduly large CBO, which acted as a barrier to carriers and reduced the current, as expected.

Following the bandgap and work function optimization of Zn(O,S) buffer, the thickness of Zn(O,S) buffer was controlled from 5 to 30 nm for the best PV performance. The thickness of the ZnS passivation layer was fixed again at 9 nm, and the ZnO:ZnS cycle ratio of Zn(O,S) buffer was tied up to 6:1. The *J*–*V* curves of the STUT CIGSe solar cell with various thicknesses of Zn(O,S) layers are shown in Figure 2c and Table 1c. The changes of PV parameters are arranged in Figure S3 and Table S3 (Supporting Information). The best STUT CIGSe solar cell with 7.5‐nm‐thick Zn(O,S) had a PCE and *J*_sc_ of 11.39% and 31.55 mA cm^−2^, respectively.

To double‐check the engineered and optimized band structure of the STUT CIGSe solar cell with 9‐nm‐thick ZnS passivation and 7.5‐nm‐thick Zn(O,S) buffer, the actual band structure of Zn(O,S)/ZnS was analyzed to compare with that of CdS buffer, which is known to form a spikelike band alignment with an optimal CBO of 0.3 eV,^[^ ^33^ ^]^ as previously mentioned. The actual electrical band structure of the STUT CIGSe solar cell with Zn‐based buffer was reconstructed as shown in Figure 2d according to the optical bandgaps of the materials and scanned VBM values from optical spectra, EQE, and XPS analysis utilizing in situ etching by Ar^+^ ion step by step. The detailed measurement results are shown in Figure S4 (Supporting Information). The difference in the VBMs of Zn(O,S) and ZnS was noticeable, which became the reference point for locating the Zn(O,S)/ZnS interface. The CBO in the Zn(O,S)/ZnS/CIGSe solar cell structure from the reconstructed band structure was identified to be ≈0.3 eV, which is also known as the optimal CBO of 0.3 eV,^[^ ^33^ ^]^ as in CdS/CIGSe.

### 2.2 Structural Analysis of STUT CIGSe Solar Cell

**Figure 3:**
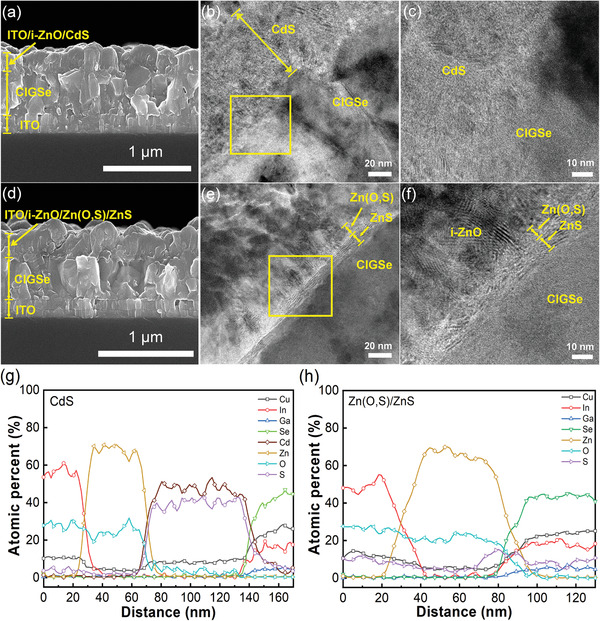
Cross‐sectional SEM images of ST CIGSe solar cells with a) CdS and d) Zn(O,S) buffer layers. TEM images of the CIGSe/buffer interface in the b,c) CdS and e,f) Zn(O,S) buffer layers. TEM line scan of the CIGSe/buffer interface in the g) CdS and h) Zn(O,S) buffer layers.

The STUT CIGSe solar cell structures with CdS and Zn‐based buffer layers were assured by cross‐sectional scanning electron microscopy (SEM) and transmission electron microscopy (TEM) analyses. **Figure** **3**a,d shows SEM images, and Figure 3b,c and e,f show TEM images of the interfaces of CdS/CIGSe and Zn(O,S)/ZnS/CIGSe, respectively. The SEM images show a rear‐side indium‐doped tin oxide (ITO) contact with a thickness of 200 nm and a CIGSe absorber of ≈500 nm. The full structure thicknesses were ≈1 µm for both structures without Al grids. The TEM images of the CdS/CIGSe interface were slightly vague, although the CdS layer thickness was still observable to be ≈60–70 nm, as shown in Figure 3b. The TEM images of the Zn(O,S)/ZnS/CIGSe interface in Figure 3e clearly show Zn(O,S)/ZnS layers with a thickness of 16.5 nm, although each of the layers was unidentifiable due to the resolution of the image. However, Moire fringe patterns^[^ ^52^, ^53^ ^]^ appeared in the ZnS passivation layer due to repeated layer‐by‐layer ALD deposition, as shown in Figure 3e,f, which differentiated the ZnS passivation layer from the Zn(O,S) buffer layer. To ensure the presence of Zn‐based structures, including ZnS passivation layers and Zn(O,S) buffers, TEM line scans were conducted across the buffer/CIGSe interface, as shown in Figure 3g,h. Again, the thickness of the CdS buffer was identified to be ≈60–70 nm based on the Cd and S profiles. On the other hand, the composition and thickness of ZnS and Zn(O,S) layers were roughly identified from the S and O profiles. As obvious from those profiles, ZnS and Zn(O,S) can be separately identified because the O profile crosses between those two peaks of the S profile. Nonetheless, long tails of the O profile at the interfaces ranging from the Zn(O,S) buffer to the top of the CIGSe absorber were observed, which may be due to the extremely thin ZnS and Zn(O,S) layers, the relatively large roughness of the CIGSe absorber surface, or the spatial resolution of energy‐dispersive X‐ray spectroscopy (EDS).^[^ ^54^, ^55^ ^]^

### 2.3 Monofacial PV Performance of STUT CIGSe Solar Cell

**Figure 4:**
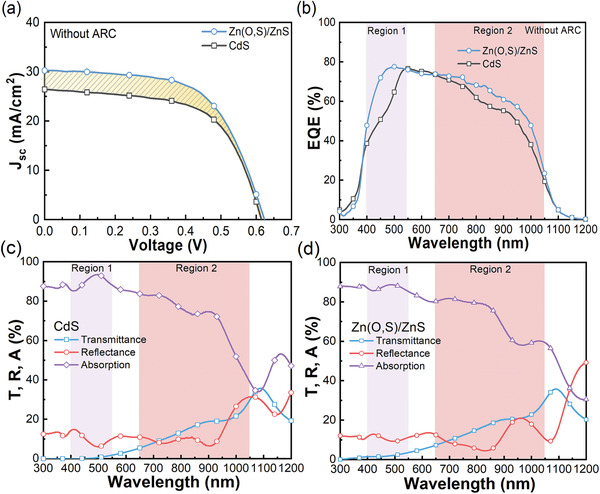
a) *J*–*V* curves (at 1 sun illumination on front‐side only), b) EQE spectra, and optical spectra of STUT CIGSe solar cells with c) CdS or d) Zn(O,S) buffers.

To investigate the PV performance enhancements of STUT CIGSe solar cells with ZnS passivation and Zn(O,S) buffer, the device with Zn‐based buffer was compared with a conventional device with a 60‐nm‐thick CdS buffer. Since STUT CIGSe solar cells suffer from low *J*_sc_ due to deficient light absorption in the ultrathin CIGS absorber layer and optical loss at front and back contact,^[^ ^56^ ^]^ the adoption of a wider bandgap buffer helped to increase the *J*_sc_ values. For the device with Zn‐based buffer, a ZnS passivation thickness of 9 nm and Zn(O,S) buffer thickness of 7.5 nm were utilized, which were optimized and confirmed previously. The best PCE of the STUT CIGSe solar cell with Zn‐based buffer was 11.62%, while that with CdS buffer was 9.88%. The major factor of PCE enhancement was the increase in *J*_sc_, whereas *V*_oc_ and FF were similar in both samples, as shown in **Figure** **4**a. The change in *J*_sc_ was an ≈17% differential increase, which must be due to the employment of wide bandgap Zn(O,S) instead of CdS buffer, in other words, due to the increased optical budget. To verify the effect of the wide bandgap, the spectral EQEs were measured and compared, as shown in Figure 4b. Two spectral regions of EQE enhancement were noticeable, as shaded by Regions 1 and 2. The EQE difference in Region 1 explicitly showed elongated incoming light into the absorber near the ultraviolet (UV) region, which contributed to the collection of carriers. The bandgap of CdS buffer is 2.4 eV, which limits the incoming light below 520 nm in wavelength, as previously mentioned, while that of Zn(O,S) is 3.14 eV, whose limitation was pushed down below 395 nm. In addition, the optimized thickness of the ZnS passivation and Zn(O,S) buffer was only 16.5 nm, which is only 27.5% of the thickness of the CdS buffer, which guarantees an increased optical budget. The longer wavelength region EQE response in Region 2 near‐infrared (NIR) also showed a wide spectral range improvement in STUT CIGSe solar cells with Zn‐based buffer. The EQE difference in Region 2 was supposedly not the issue of rear‐side recombination or barriers because both samples were fabricated in the same batch until CIGSe deposition. Hence, the optical properties of STUT CIGSe solar cells with CdS or Zn‐based buffer were required to investigate the cause of the difference.

The optical spectra were measured by UV–vis–NIR spectroscopy, as shown in Figure 4c,d. The averaged transmittances in the wavelength range of 380–780 nm of the devices with CdS‐ or Zn‐based buffer were 3.89% and 5.57%, respectively. In addition, the average visible transmittances (AVTs) calculated depending on the photopic response of human eye^[^ ^57^ ^]^ were 2.25% and 4.00%, respectively. The transmittance (*T*) profiles were very similar in the two devices except for the higher transparency near the UV region in the device with Zn‐based buffer. On the other hand, the reflectance (*R*) and absorption (*A*) of the two devices were different from each other. One of the apparent differences is the spectral reflectance response in the range of ≈700–1100 nm because optical spectra longer than 1100 nm were out of focus since the CIGSe absorption edge was up to ≈1080 nm due to the bandgap of 1.18 eV. The reflectance ripples of the device with Zn‐based buffer fluctuated almost reversely to those with CdS buffer, which can be attributed to the optical interferences based on the buffer's thickness and refractive index differences. In particular, the spectral refractive index difference of CdS and Zn‐based buffer may lead to the spectral reflectance difference, which resulted in an overall reduction in reflectance in Region 2. The refractive indices of CIGSe, CdS, ZnS, and ZnO are ≈2.90, 2.43, 2.39, and 1.61, respectively,^[^ ^58^, ^59^ ^]^ although there are slight differences due to the calculation or measurement approach among researchers. The refractive index of ALD‐grown Zn(O,S) differs and is reported to vary between ZnO and ZnS according to the composition of oxygen and sulfur.^[^ ^59^ ^]^ The other explanation for the reduction in reflectance is that the Zn‐based buffer consisted of double layers, which may lead to a relatively gradual increase in the refractive index. The CdS buffer consisted of a single layer comparatively, which may provide a relatively abrupt change in the refractive index. Hence, the relatively gradual increase may have led to a lower reflection. The reduced reflectance of the device with Zn‐based buffer directly increased the absorption spectra because the transmittance spectra were similar in this region. Thus, the difference in EQE in Region 2 can be understood as one of the effects of substitution from CdS to Zn‐based buffer in the STUT CIGSe solar cell structure. The increase in EQE contributed to the *J*_sc_ increase in the solar cell device with Zn‐based buffer and continued to enhance the PV performance of the STUT CIGSe solar cell with Zn‐based buffer. As shown in **Table** **2**, *J*_sc_ increased by ≈17% after substituting CdS with Zn‐based buffer, and the PCE increased as much, accordingly.

**Table 2:** PV parameters of STUT CIGSe solar cells with different buffers

Sample	*V*_oc_ [V]	*J*_sc_ [mA cm^−2^]	FF [%]	PCE [%]
CdS	0.6147	26.42	60.84	9.88
Zn(O,S)/ZnS	0.6223	30.91	60.42	11.62

### 2.4 Bifacial PV Performance of STUT CIGSe Solar Cell

Thus far, the STUT CIGSe solar cell with Zn‐based buffer has been investigated from the point of monofacial application. The introduction of Zn‐based buffer instead of CdS enlarged the optical budget and increased the incoming light into the absorber by utilizing the larger bandgaps of Zn‐based materials compared to CdS. As expected, the increased optical budget obtained by employing a wider bandgap buffer majorly increased the *J*_sc_ and PCE in turn. In addition, there can be more methods to further enlarge the optical budget. Two more methods were considered in this part. One of them is to apply an ARC on top of the STUT CIGSe solar cell, and the other is literally to increase the amount of incoming light by more than 1 sun, such as a bifacial application. The ARC reduces the reflection of the incoming light, improving the absorption of the solar cell, and the bifacial solar cell application receives 1 sun from the front‐side illumination and additionally receives 0.2–0.3 sun^[^ ^37^, ^38^ ^]^ from the rear‐side illumination depending on the practical installation sites based on the albedo effect. In this regard, the STUT CIGSe solar cell has another merit since it can be applied directly as a bifacial application without any change in the stacking structure, which is a different utilization of the optical budget from Si‐based bifacial solar cells without semitransparency^[^ ^60^, ^61^ ^]^ and an additional advantage of the STUT solar cell in comparison. However, there can be a gap in the expected power generation with the difference in PCEs of front‐ and rear‐side illumination conditions because the PCE under monofacial illumination varies depending on the illumination direction.^[^ ^62^, ^63^ ^]^

**Figure 5:**
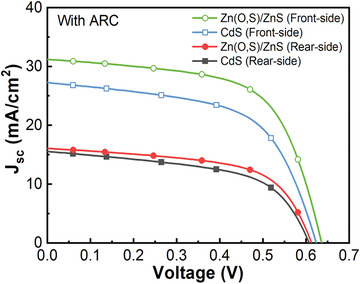
*J*–*V* curves of STUT CIGSe solar cells with different buffers after ARC under rear‐ or front‐side 1 sun illumination.

For this reason, to investigate the effect of ARC under single‐side illumination and further the effect of bifacial application based on the previous optimization results with wide bandgap Zn‐based buffer structure, a new set of STUT CIGSe solar cell samples were prepared with CdS or Zn‐based buffer. The PCEs before ARC of those newly prepared samples were 9.42% for the STUT CIGSe solar cell with CdS buffer, and 11.37% for that with Zn‐based buffer, which are very similar to the best PCE, respectively. After ARC, both the PCE of STUT CIGSe solar cells with CdS and Zn‐based buffer increased under front‐side 1 sun illumination, as shown in **Figure** **5** and **Table** **3**. The PCE of the STUT CIGSe solar cell with CdS buffer increased from 9.42% to 10.03% due to the ARC, while that with Zn‐based buffer increased from 11.37% to 12.41%. As expected, the major factor of improvement in the solar cell with CdS buffer was the increase of *J*_sc_ and that in the solar cell with Zn‐based buffer was the increases of *J*_sc_ and FF, while the *V*_oc_ was also slightly increased because of the *J*_sc_ and *V*_oc_ relationship from Equation (1).^[^ ^64^ ^]^

(1) $$V_{\mathrm{oc}}=\frac{AKT}{e}\;\cdot \ln\left(\frac{J_{\mathrm{sc}}}{J_{0}}\right)$$

where *A, K, T, e*, and *J*_0_ are the diode ideality factor, Boltzmann's constant, measurement temperature, electronic charge, and saturation current, respectively. Thus, the ARC also provided an additional increase in the optical budget of STUT CIGSe solar cells, especially in the STUT CIGSe solar cell with Zn‐based buffer. On the other hand, under rear‐side 1 sun illumination, both PCEs of STUT CIGSe solar cells with CdS and Zn‐based buffer were almost halved, mainly due to the decrease in *J*_sc_. In addition, *V*_oc_ and FF were also decreased slightly in both cell lines. The PCE difference depending on illumination direction is typically observable due to the generated location and diffusion lengths of carriers, the structure‐dependent charge collection efficiency, the back contact recombination, and so on.^[^ ^9^, ^63^ ^]^ Nonetheless, all PV parameters of the STUT CIGSe solar cell with Zn‐based buffer were relatively superior to those with CdS buffer. However, the relative superiority was reduced under rear‐side illumination. Therefore, the illumination direction should be considered for the best utilization of STUT CIGSe solar cells in bifacial applications, and it is roughly expected that the power generation of bifacial STUT CIGSe solar cells will increase close to 1.5 times of the monofacial power generation when the sum of the incoming light is 2 sun (i.e., each 1 sun from the front‐ and rear‐side illumination).

**Table 3:** PV parameters of STUT CIGSe solar cells with different buffers after ARC under rear‐ or front‐side 1 sun illumination

STUT CIGSe solar cell	Illumination direction (1 sun)	*V*_oc_ [V]	*J*_sc_ [mA cm^−2^]	FF [%]	PCE [%]
With CdS buffer	Rear	0.6072	15.56	56.62	5.35
	Front	0.6237	27.26	59.02	10.03
With Zn(O,S)/ZnS buffer	Rear	0.6127	16.07	60.25	5.93
	Front	0.6363	31.22	62.47	12.41

To provide and investigate an even higher optical budget for STUT CIGSe solar cells, a bifacial application based on the albedo effect was considered. The bifacial application of the STUT CIGSe solar cell was investigated by measuring the PV properties under bifacial illumination in the bifacial measurement setup, which enables double‐sided illumination without bias. Bifacial illumination was considered to encompass not only the illumination from the front side but also that from the rear side, which can be reflected from the ground, air, object, exterior of building,^[^ ^62^ ^]^ including application cases of indoor, window, and outdoor environments. Hence, as previously defined and introduced, BCE was utilized to represent the PV performance of bifacial semitransparent solar cells with the seamless extension of PCE and reflect the additional power generation under practical bifacial conditions (i.e., 1.0 sun from the front‐side illumination with 0.0–1.0 sun from the rear‐side coincidental illumination due to the albedo effect, which corresponds to a total incoming light of 1.0–2.0 sun).

**Figure 6:**
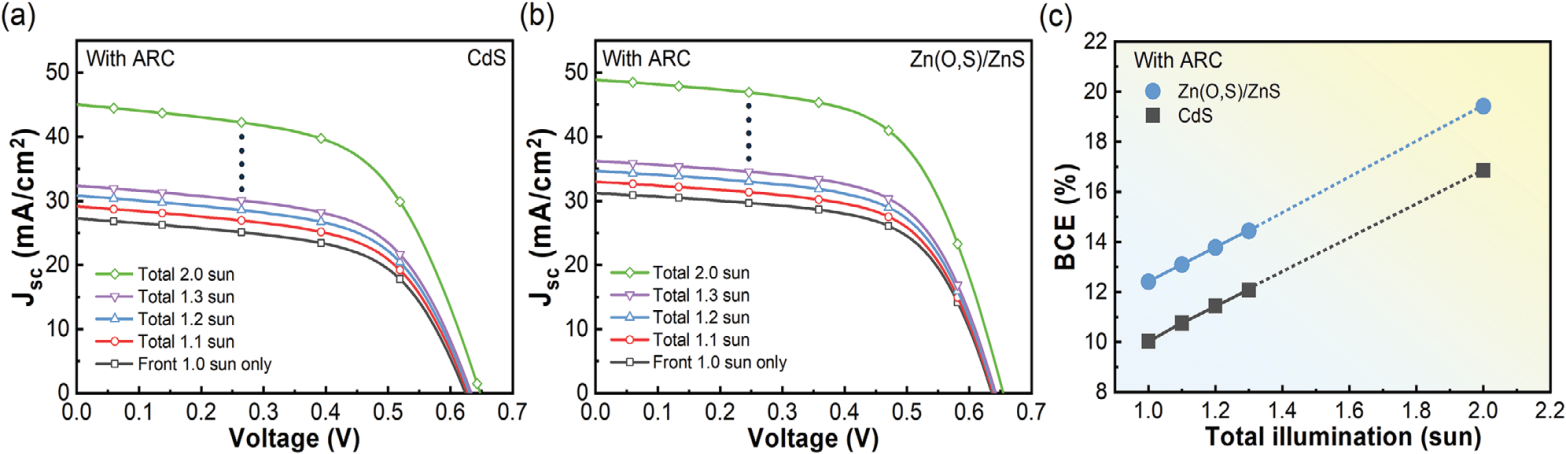
*J*–*V* curves of AR‐coated bifacial STUT CIGSe solar cells with a) CdS‐ or b) Zn‐based buffer under bifacial illumination (the front‐side illumination was fixed at 1.00 while the rear‐side coincidental illumination changed from 0 to 1.0 sun), and c) the variation in BCE according to the increase in total incoming light.

As the rear‐side illumination was gradually increased from 0.0 to 1.0 sun while fixing the front‐side illumination at 1 sun (i.e., total 1.0–2.0 sun), *J*–*V* curves and BCEs were measured, as shown in **Figure** **6** and **Table** **4**. The measured *J*_sc_ values of bifacial STUT CIGSe solar cells with CdS‐ or Zn‐based buffer almost linearly increased as the rear‐side illumination increased linearly, while the *V*_oc_ and FF changes remained at similar levels. Additionally, as explicit and direct results, the BCEs increased linearly, as shown in Figure 6c. Not only for a general 20–30% albedo but also over the whole range of 1–2 sun of total illumination, the STUT CIGSe solar cell with Zn‐based buffer exhibited relatively superior PCE and BCEs, and the efficiency difference gap between the bifacial STUT CIGSe solar cell with Zn‐based buffer and that with CdS buffer slightly increased as the rear‐side illumination increased. At a total illumination of 1.3 sun, the BCEs of bifacial STUT CIGSe solar cells with CdS and Zn(O,S) buffer layer were 12.08% and 14.44%, respectively. It was interesting to observe that the best BCE of bifacial STUT CIGSe solar cell with CdS buffer measured as 16.85% and that of bifacial STUT CIGSe solar cell with Zn‐based buffer further achieved 19.42% BCE at total illumination of 2 sun, which were almost exactly on the extrapolated line of BCE expectation based on the increased illumination. The BCE of 19.42% under 2 sun (1 sun illumination on each side) physically means that the bifacial cell under 2 sun can produce the equivalent power output to a monofacial cell with 19.42% PCE (under 1 sun). Hence, as demonstrated by Figure 6c, the bifacial STUT CIGSe solar cell can take advantage of the albedo effect and the enlarged optical budget of the bifacial STUT CIGSe solar cell directly increases *J*_sc_, which increases the BCE in series.

**Table 4:** PV parameters of AR‐coated bifacial STUT CIGSe solar cells with CdS‐ or Zn‐based buffer under bifacial illumination (the front‐side illumination was fixed at 1.00, while the rear‐side coincidental illumination changed from 0 to 1.0 sun)

Buffer layer	Total Illumination	*V*_oc_ [V]	*J*_sc_ [mA cm^−2^]	FF [%]	BCE [%]
CdS	Front 1.0 sun only	0.6237	27.26	59.02	10.03
	Total 1.1 sun (Front: 1.0 sun + Rear: 0.1 sun)	0.6276	29.14	58.86	10.76
	Total 1.2 sun (Front: 1.0 sun + Rear: 0.2 sun)	0.6307	30.81	58.88	11.44
	Total 1.3 sun (Front: 1.0 sun + Rear: 0.3 sun)	0.6337	32.36	58.92	12.08
	Total 2.0 sun (Front: 1.0 sun + Rear: 1.0 sun)	0.6479	45.02	57.78	16.85
Zn(O,S)/ZnS	Front 1.0 sun only	0.6363	31.22	62.47	12.41
	Total 1.1 sun (Front: 1.0 sun + Rear: 0.1 sun)	0.6378	32.99	62.22	13.09
	Total 1.2 sun (Front: 1.0 sun + Rear: 0.2 sun)	0.6402	34.66	62.12	13.78
	Total 1.3 sun (Front: 1.0 sun + Rear: 0.3 sun)	0.6426	36.24	62.00	14.44
	Total 2.0 sun (Front: 1.0 sun + Rear: 1.0 sun)	0.6546	48.87	60.72	19.42

**Figure 7:**
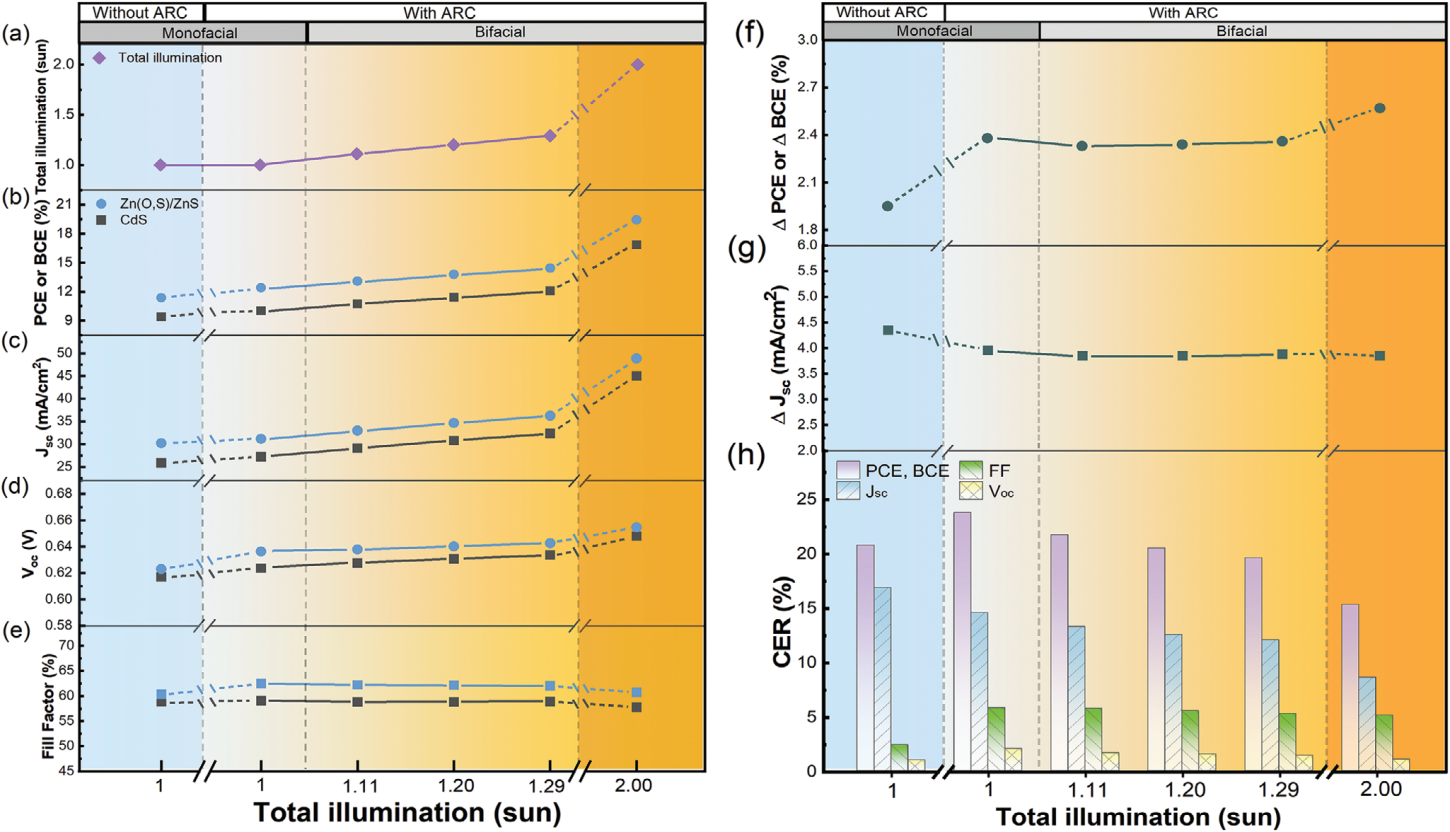
PV parameters and comparisons of bifacial STUT CIGSe solar cells with CdS or Zn‐based buffer with increasing total illumination. a) The increase in total illumination, b–e) PV parameters, the difference in f) PCE or BCE, g) *J*_sc_, and h) CERs between bifacial STUT CIGSe solar cells with Zn‐based buffer and those with CdS buffer, according to total illumination.

For a more detailed discussion, the previous photovoltaic parameters from the *J*–*V* curves of STUT CIGSe solar cells with CdS and Zn‐based buffer in Tables 2, 3, 4 were rearranged in **Figure** **7** according to the increase in the optical budget in Figure 7a. As clearly observable in Figure 7b, PCE and BCE increased while total illumination on both bifacial STUT CIGSe solar cells increased. Additionally, the *J*_sc_ values followed the shape of the illumination increase, while *V*_oc_ slowly increased in comparison and FF slowly decreased, as shown in Figure 7c–e, which suggested that the major reason for the BCE increase was the increase in *J*_sc_. For all of the photovoltaic parameters, the bifacial STUT CIGSe solar cell with Zn‐based buffer exhibited superior characteristics to those with CdS buffer.

Looking into detail by subtracting the parameter of the bifacial STUT CIGSe solar cell with CdS buffer from that with Zn‐based buffer reveals that the BCE difference increases slightly, as shown in Figure 7f, while the *J*_sc_ difference decreases slightly, as shown in Figure 7g. The absolute *J*_sc_ value of bifacial STUT CIGSe solar cell with Zn‐based buffer was always higher compared to that with CdS thoughout all the tested illumination conditions because the difference has a plus sign. However, the slightly increasing trend of the BCE difference with the slightly decreasing trend of the *J*_sc_ difference implies that the nanolevel thickness of Zn(O,S) may have limited the enhancement of *J*_sc_ according to the increase in illumination since our previous calibration on Zn(O,S) buffer thickness was based on 1 sun illumination, which can be different under higher illumination conditions. In other words, if proper calibrations on targeting illumination are carried out, even more enhancement in *J*_sc_ and BCE at higher illuminations can be possible in bifacial STUT CIGSe solar cells with Zn‐based buffer structures. To show the relative enhancement of the bifacial STUT CIGSe solar cell with Zn‐based buffer compared with CdS buffer, we define the comparative enhancement ratio (CER) as follows by Equation (2):

(2) $$\begin{array}{ccc} & & \mathrm{Comparative}\;\mathrm{enhanced}\;\mathrm{ratio}\;\left(\mathrm{CER}\right)\\ & & \;=\frac{PP_{\mathrm{Zn}\left(O,S\right)}\;-\;PP_{\mathrm{CdS}}}{PP_{\mathrm{CdS}}\;}\;\times \;100\;\left(\%\right) \end{array}$$

where PP represents each of the PV parameters, such as PCE, BCE, *J*_sc_, FF, or *V*_oc_.

All CERs, including PCE, BCE, *J*_sc_, FF, and *V*_oc,_ in bifacial STUT CIGSe solar cells with Zn‐based and CdS buffers were positive, which indicates the comparative enhancements of bifacial STUT CIGSe solar cells with Zn‐based buffers. In addition, the slightly decreasing trend of CER according to the increase in total illumination reflects that the enhancement gap is saturated at higher illumination conditions. This seems reasonable because the PCE is saturated with increasing total illumination, similar to the reported result in the concentrator application.^[^ ^65^ ^]^ However, between the 1 and 2 sun illumination levels, an almost linear increase in enhancements can be expected, which is an obvious advantage of providing practical and additional power generation in bifacial STUT CIGSe solar cells. Hence, more proactive studies utilizing the albedo effect may further enhance the BCE of bifacial STUT CIGSe solar cells. The CER results according to the increase in total illumination are summarized in Figure 7h.

Furthermore, it is an interesting consideration to compare (1) the respective summation of *J*_sc_ values and PCEs from separate measurements under 1 sun on single‐side illumination, as shown in Figure 5 and Table 3 (by repeating conventional monofacial measurement on the front side and rear side) and (2) the *J*_sc_ value and BCE under a total of 2 sun simultaneous double‐side illumination, as shown in Figure 6 and Table 4 (simultaneous bifacial measurement under each side 1 sun illumination). Accordingly, it is expected that those two results (i.e., the results from (1) and (2)) are similar because they have only simple differences, whether the efficiency was measured under single‐side illumination or double‐side illumination, and whether the total illumination was 1 or 2 sun. However, we found that those two results are similar but not the same, as arranged in **Table** **5**. In Table 5a, *J*_sc_ and PCE were summed, while *V*_oc_ was estimated based on Equation (1), and the FF were calculated from the other parameters. A comparison of Table 5a and b clearly shows that the summation of the separate measurements under 1 sun single‐side illumination underestimated the actual power generation which was assessed by the actual bifacial measurement under a simultaneous total of 2 sun double‐side illumination. The most dramatic differences were found in *J*_sc_ and PCE values, as expected. Previously, we expected that the power generation of bifacial STUT CIGSe solar cells would increase to roughly “more than 1.5 times” the monofacial power generation (measured under 1 sun illumination) when the sum of incoming light becomes 2 sun, and here, we confirmed that by showing the *J*_sc_ and PCE differences. One of the major reasons for these differences can be attributable to the albedo effect and dark current of the solar cell which produces parasitic power dissipation. In other words, the two separate monofacial measurements may doubly account for the dark currents, while the simultaneous bifacial measurement accounts for it only once. Therefore, in a usual circumstance where the albedo effect is valid to a degree at which the dark current can be compensated, the actual power generation capability of bifacial STUT CIGSe solar cells will surpass the expected power generation based on the PCE, which is measured under single‐side illumination. Subsequently, it also demonstrates the necessity of the “BCE” definition for the exact surplus power generation of bifacial solar cells based on the albedo effect. In addition, the largest percentile difference among all PV parameters in Table 5 between the single‐side illumination measurement and the double‐side illumination one is observed in *J*_sc_ of the solar cell with CdS buffer, where the relatively higher *J*_sc_ difference in the solar cell with CdS buffer compared to that in the solar cell with Zn(O,S) buffer can be explained by 8 times thickness difference of the buffer layers, which are 60 nm for CdS and 7.5 nm for Zn(O,S), respectively. Therefore, an additional thickness optimization of Zn(O,S) buffer according to the total illumination may further enhance the BCE of STUT CIGSe solar cell with Zn(O,S) buffer if the targeting illumination is fixed considering the environmentally specific albedo. Further detailed investigations on the dark current and effect on BCE may provide a greater chance to enhance the power generation ability of bifacial STUT CIGSe solar cells.

**Table 5:** a) *J*_sc_ and PCE summation of STUT CIGSe solar cells with different buffers under 1 sun rear‐ or front‐side illumination (calculated from Table 3) and b) *J*_sc_ and BCE under a total of 2 sun double‐side illuminations (excerpted from Table 4)

Bifacial STUT CIGSe solar cell (with ARC)	(a) Summation of separate measurements under single‐side illumination (1 sun)				(b) Simultaneous measurement under double‐side illumination (total 2 sun)			
	*V*_oc_^a)^ [V]	Sum of *J*_sc_ [mA cm^−2^]	FF^a)^ [%]	Sum of PCE [%]	*V*_oc_ [V]	*J*_sc_ [mA cm^−2^]	FF [%]	BCE [%]
With CdS buffer	0.6366	42.82	56.42	15.38	0.6479	45.02	57.78	16.85
With Zn(O,S) buffer	0.6488	47.29	59.77	18.34	0.6546	48.87	60.72	19.42

^a)^ These values were estimated based on dark *J*–*V* results and Equation (1), only for comparison.

## 3 Conclusion

The design of the STUT CIGSe solar cell started with a consideration of monofacial application with semitransparency not only for energy harvesting but also for architectural components. The most popular CIGSe solar cell structure generally uses CdS buffer because of the advantages in bandgap alignment and high PCE achievement. However, the limited optical budget of incoming light inevitably compels the trade‐off between PCE and semitransparency, making the employment of a wide bandgap buffer crucial to increase the amount of incoming light into the absorber. Hence, the wider bandgap opening of the buffer to incoming light becomes a major factor to increase the *J*_sc_ and PCE of the STUT CIGSe solar cell. To fundamentally increase the optical budget of incoming light and increase the PCE and BCE in STUT CIGSe solar cells, several approaches were attempted step by step. The start was CIGSe surface passivation by a wide bandgap ZnS nanolayer. ZnS passivation improved the junction quality by reducing the diode ideality factor from 2.025 to 1.191, reducing the diode leakage current density by approximately two orders of magnitude and increasing the shunt resistance from 331.7 to 1299 Ω cm^2^ compared with the sample without passivation. The second approach was the adoption of Zn(O,S) buffer, which fundamentally enlarged the optical budget. The *J*_sc_ of the STUT CIGSe solar cell with Zn(O,S)/ZnS buffer increased by ≈17% compared with that with CdS buffer, while the *V*_oc_ and FF also increased slightly. The differential increase in *J*_sc_ by the adoption of Zn(O,S) enabled a substantial enhancement in the PCE. The third was ARC to enlarge the optical budget by reducing reflection on the surface of the STUT CIGSe solar cell, which resulted in an additional *J*_sc_ increase. The PCE of the STUT CIGSe solar cell with a Zn‐based buffer structure after ARC reached 12.41%. Further enlargement of the optical budget was pursued by simulating the albedo effect utilizing a bifacial measurement setup. The bifacial performances were tested with total illumination from 1.1 to 2.0 sun by increasing the rear‐side illumination from 0.1 to 1.0 sun while fixing the front‐side illumination at 1 sun. The BCE of the STUT CIGSe solar cell with a Zn‐based buffer structure reached 14.44% at a total illumination of 1.3 sun, and the BCE finally reached 19.42% at 2.0 sun. Therefore, in this sense, the replacement of the buffer layer by an even wider material such as SnO_2_, ZnTiO, ZnSnO, or ZnMgO may further extend the optical budget of bifacial STUT CIGSe solar cells. In addition, by comparing the summation of the separate measurements under 1 sun single‐side illumination and the bifacial measurement under a simultaneous total 2 sun double‐side illumination, we found that the former may underestimate the actual power generation with the albedo effect. More pioneering studies on designing a novel bifacial STUT CIGSe solar cell (or module) structure combined with internal or external reflectors and making an utmost use of the albedo effect may further enhance the BCE of STUT CIGSe solar cells, surpass the economic feasibility, and accelerate the expansion of the BIPV and BISTPV market. In the future, the results of this work on STUT CIGSe solar cells can also be utilized for bifacial applications and in other semitransparent PVs, smart windows, top cells of tandem structures, and so on.

## 4 Experimental Section

### Preparation of the STUT CIGSe Absorber and Solar Cell Structure

**Figure 8:**
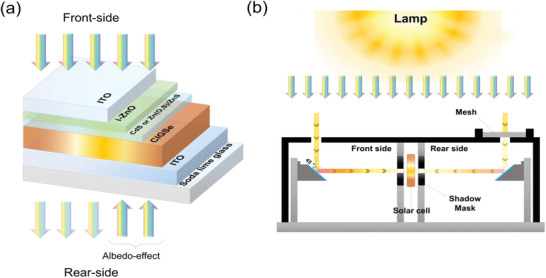
Schematics of a) STUT CIGSe solar cell structure with different buffer layers and b) bifacial cell tester.

Before depositing the STUT CIGSe thin film, the 1.1‐mm‐thick soda‐lime glass substrates with 200‐nm‐thick ITO bottom electrode on top were cleaned by a series of acetone, alcohol, and deionized (DI) water baths for 15 min each. After drying, the substrates were loaded into a coevaporation chamber, which was pumped down to a pressure of 1 × 10^−6^ mTorr. The substrates were heated to a deposition temperature of 500 °C. Then, a STUT CIGSe absorber layer with a thickness of 500 ± 20 nm was deposited for 24 min by a single‐stage coevaporation process using Cu, In, Ga, and Se effusion cells. Subsequently, the samples were cooled to 400 °C in a Se atmosphere for PDT, and NaF PDT was performed for 10 min in situ, under which the optimal conditions were calibrated in a previous study.^[^ ^19^ ^]^ The EDS analyses of the STUT CIGSe absorber indicated a Cu/(Ga + In) of 0.75–0.80 and a Ga/(Ga + In) of 0.25–0.28, which resulted in a CIGSe absorber bandgap of 1.18 eV. The PDT‐processed STUT CIGSe absorber was followed by CBD of ≈60‐nm‐thick CdS as a buffer layer, sputtering of 40 to 50‐nm‐thick i‐ZnO as a window layer, and sputtering of 150‐nm‐thick ITO as the front electrode. In case of STUT CIGSe solar cells with Zn‐based buffer, ALD depositions of ZnS, Zn(O,S), and i‐ZnO were followed instead of CBD CdS buffer and sputtering i‐ZnO. Finally, a 1‐µm‐thick Al grid and 110‐nm‐thick MgF_2_ ARC were deposited by thermal evaporation. The isolated area of the STUT CIGSe solar cell except the Al grid was ≈0.42 cm^2^ and each cell area was exactly calibrated including the front and rear‐side, respectively. A detailed schematic of the STUT CIGSe cell is shown in **Figure** **8**a.

### ALD Deposition of ZnS Passivation Layer, Zn(O,S) Buffer, and i‐ZnO Window Layer

The deposition temperature and working pressure of the ALD process were fixed at 100 °C and 1.5 Torr for ZnS, Zn(O,S), and i‐ZnO layer. The precursor or reactor materials for zinc, oxygen, and sulfur were diethylzinc (DEZn: Zn(C_2_H_5_)_2_), DI water, and diluted hydrogen sulfide (H_2_S 5%), respectively. Argon (99.999%) was used as the carrying and purging gas. The experimental processes of ZnS and ZnO deposition were conducted in order. The process cycle for ZnS was precursor (DEZn), purge (Ar), reactor (H_2_S), and purge (Ar), and for ZnO, precursor (DEZn), purge (Ar), reactor (H_2_O), and purge (Ar). The pulse times of the precursor and reactors were 0.3 s (DEZn), 2.0 s (H_2_S), and 0.3 s (H_2_O), respectively. The process of Zn(O,S) deposition was organized by repeating the cycles of ZnO and ZnS with a controlled number of repetitions. To attain the best CBO in the STUT CIGSe solar cell, the Zn(O,S) buffer layer was calibrated to have a CBO value of ≈0.3 eV, where the ZnO:ZnS cycle ratio was 6:1 and the atomic ratio of [S]/([S]+[O]) in Zn(O,S) buffer was equal to 0.24 throughout this report. A more detailed procedure of ALD deposition can be found in the previous work.^[^ ^29^ ^]^

### Characterization of Thin Films and STUT CIGSe Solar Cells

Ultrathin CIGSe absorber layers and STUT CIGSe solar cells with different buffer layers were analyzed using various characterization methods. To anticipate and design the interfaces of the buffer layer and ultrathin CIGSe absorber, the optical, electrical, and compositional properties were investigated using UV–vis–NIR spectrophotometry (UV‐2600, Shimadzu, Japan) and XPS (K‐Alpha+, Thermo Fisher Scientific, United States). The morphological and compositional properties of ultrathin CIGSe absorbers were observed by SEM and EDS (S‐4700, Hitachi, Japan), respectively. The interface of CIGSe/buffer was investigated via TEM (JEM‐ARM200F, JEOL, Japan). Coupled TEM‐EDS was utilized to observe the compositional line scans. The photovoltaic parameters of STUT CIGSe solar cells were acquired via *J*–*V* measurement under a 1‐sun (AM1.5G) spectrum with an intensity of 100 mW cm^−2^ using a solar simulator (K201‐LAB 50, McScience, Republic of Korea) with a source meter (2440 5A, Keithley, United States). The spectral EQE profiles were measured based on the wavelength‐dependent carrier collection efficiency (S‐9203 HINODE mini 5, Soma, Japan).

### Measurements of Bifacial STUT CIGSe Solar Cells

Bifacial PV parameters of STUT CIGSe solar cells were determined using another solar simulator (SAN‐EI ELECTRIC CO., LTD, model: XHS‐220S1, Japan) with a bifacial cell tester for double‐side illumination.^[^ ^66^ ^]^ The class‐AAA flash simulator (xenon and halogen lamp) was utilized as the light source to ensure stability for the cell at 25 °C with temporal stability and spatial uniformity in the total area of the tester (up to 220 × 220 mm^2^) lower than 2%. The bifacial cell tester was finished with black matt paint and consisted of two mirrors placed symmetrically at 45°. Utilizing the bifacial cell tester for a bifacial measurement, the solar simulator setup provided both front‐side 1 sun (AM 1.5G) illumination and rear‐side 0–1 sun at the same time. The intensity of the rear‐side illumination was controlled by selecting an appropriate mesh to provide 0.1, 0.2, and 0.3 sun according to the standard IEC TS 60904‐1‐2. A detailed schematic of the bifacial cell tester is shown in Figure 8b.

## Conflict of Interest

The authors declare no conflict of interest.

## Supporting information

Supporting Information

## Data Availability

The data that support the findings of this study are available from the corresponding author upon reasonable request.
