# Supplementary material for: Practical Enhancements in Current Density and Power Generation of Bifacial Semitransparent Ultrathin CIGSe Solar Cells via Utilization of Wide Bandgap Zn‐Based Buffer
Source: Adv Sci (Weinh). 2022 Feb 23;9(13):2105436. doi: 10.1002/advs.202105436 (PMC9069376; doi:10.1002/advs.202105436)
Supplement: Supplementary file 1 — Supporting Information [file ADVS-9-2105436-s001.pdf]

WILEY-VCH

ADVANCED SCIENCE
Open Access

Supporting Information

for *Adv. Sci.*, DOI: 10.1002/advs.202105436

Practical Enhancements in Current Density and Power Generation of Bifacial Semitransparent Ultrathin CIGSe Solar Cells via Utilization of Wide Bandgap Zn-Based Buffer

*Dongryeol Kim, Sang Su Shin, Yonghee Jo, Sang Min Lee, Seung Kyu Ahn, Jun-Sik Cho, Jae Ho Yun, Ho Seong Lee, and Joo Hyung Park**

Supporting Information

Practical Enhancements in Current Density and Power Generation of Bifacial Semitransparent Ultrathin CIGSe Solar Cells via Utilization of Wide Bandgap Zn-Based Buffer

Dongryeol Kim, Sang Su Shin, Yonghee Jo, Sang Min Lee, Seung Kyu Ahn, Jun-Sik Cho, Jae Ho Yun, Ho Seong Lee, and Joo Hyung Park[*]

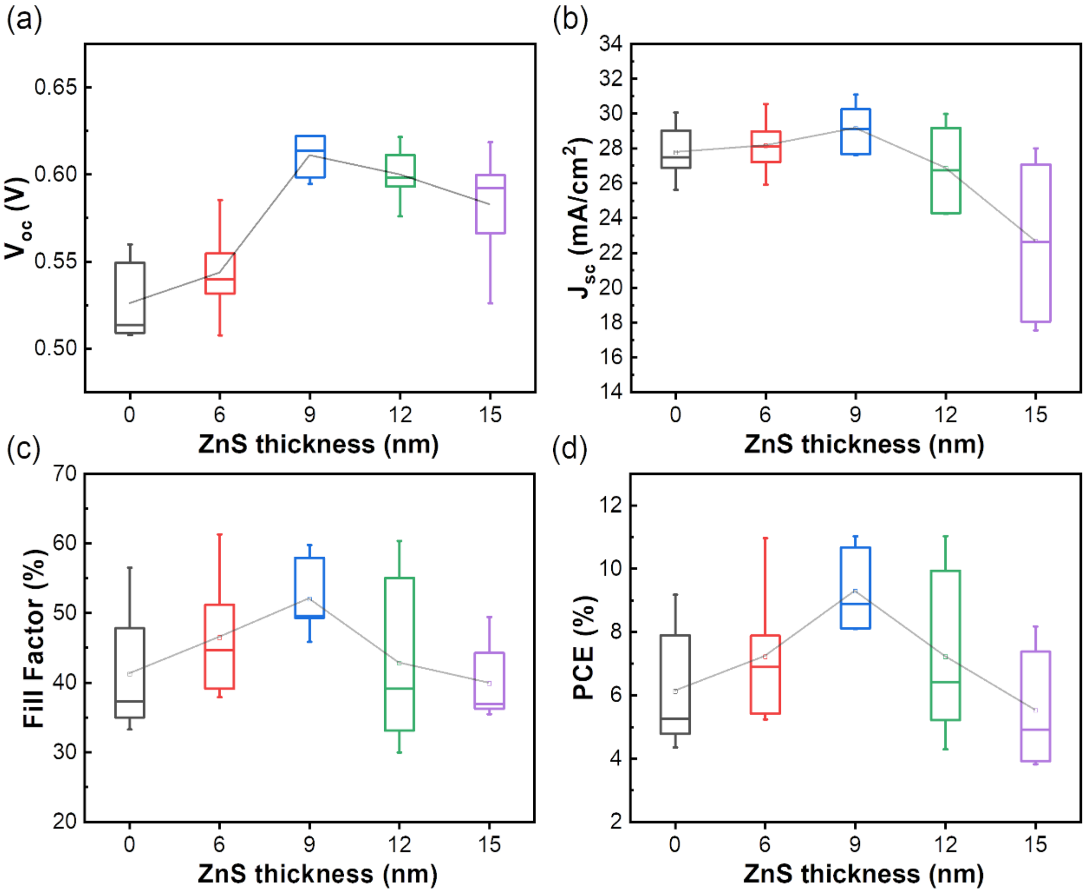

Figure S1. The distribution of PV parameters in STUT CIGSe solar cells with different thicknesses of ZnS passivation layers (total 30 solar cells). Lower and upper ends of the box indicate 25 and 75% values, whereas upper and lower whiskers located at extreme ends of each distribution represent the highest and lowest values, respectively.

Table S1. Summary of the PV properties in STUT CIGSe solar cells with different thicknesses of ZnS passivation layers (total 30 solar cells) showing the average and standard deviation.

ZnS layer [nm]	V_{oc} [V]	J_{sc} [mA/cm^2]	FF [%]	PCE [%]
0	0.5255±0.023	27.75±1.607	41.21±9.048	6.121±1.945
6	0.5431±0.026	28.14±1.576	46.47±8.719	7.216±2.091
9	0.6108±0.012	29.14±1.451	52.01±5.520	9.285±1.343
12	0.5997±0.016	26.86±2.647	42.78±12.19	7.220±2.674
15	0.5825±0.032	22.66±5.054	39.88±5.668	5.521±1.897

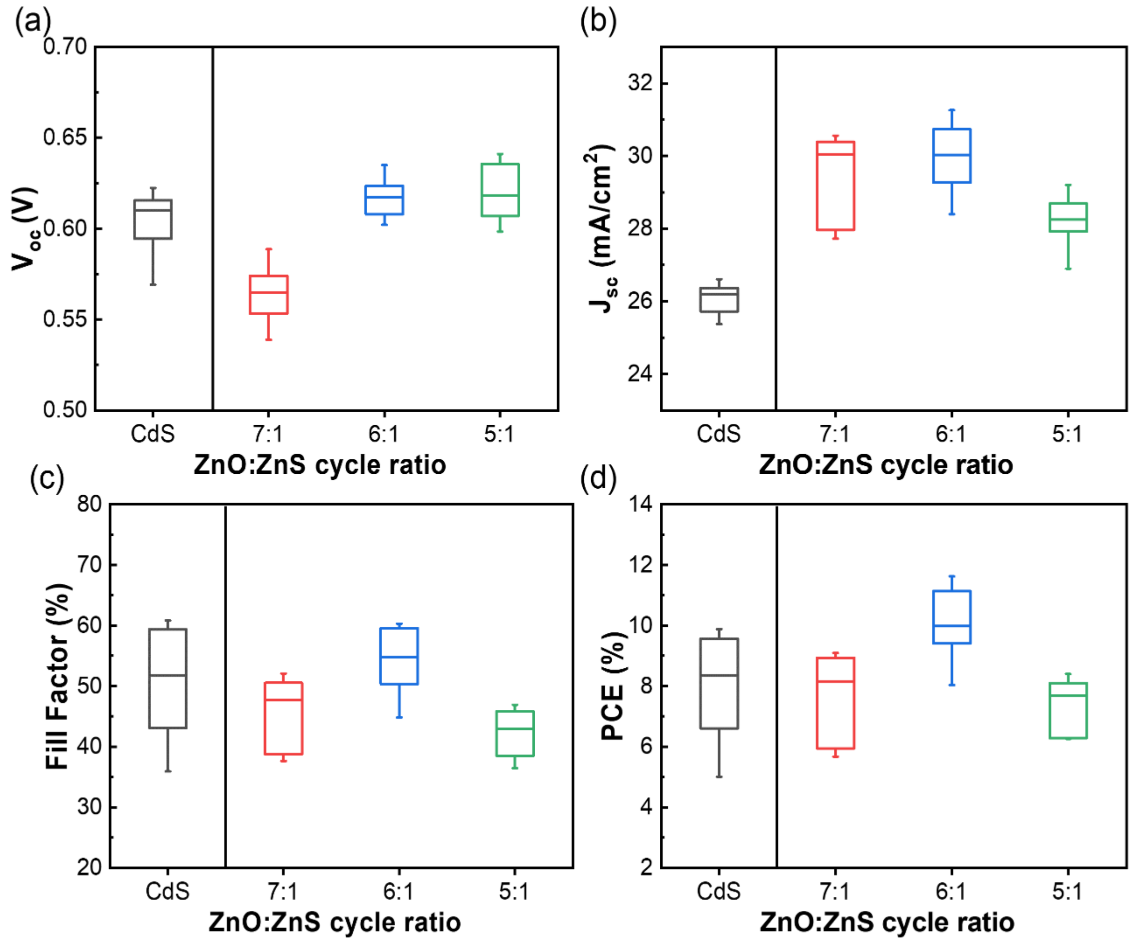

Figure S2. The distribution of PV parameters in STUT CIGSe solar cells with different ZnO to ZnS cycle ratio of Zn(O,S) buffer layers (total 36 solar cells). Lower and upper ends of the box indicate 25 and 75% values, whereas upper and lower whiskers located at extreme ends of each distribution represent the highest and lowest values, respectively.

Table S2. Summary of the PV properties of STUT CIGSe solar cells with different ZnO to ZnS cycle ratio of Zn(O,S) buffer layers (total 36 solar cells) showing the average and standard deviation.

Zn(O,S) [ZnO:ZnS cycle ratio]	V_{oc} [V]	J_{sc} [mA/cm^2]	FF [%]	PCE [%]
CdS	0.6047±0.015	25.97±0.5971	50.73±8.690	8.020±1.644
7:1	0.5640±0.017	29.45±1.267	45.72±6.089	7.657±1.489
6:1	0.6178±0.011	29.96±0.8878	53.95±6.132	10.01±1.314
5:1	0.6196±0.016	28.20±0.7823	42.25±4.255	7.400±0.9422

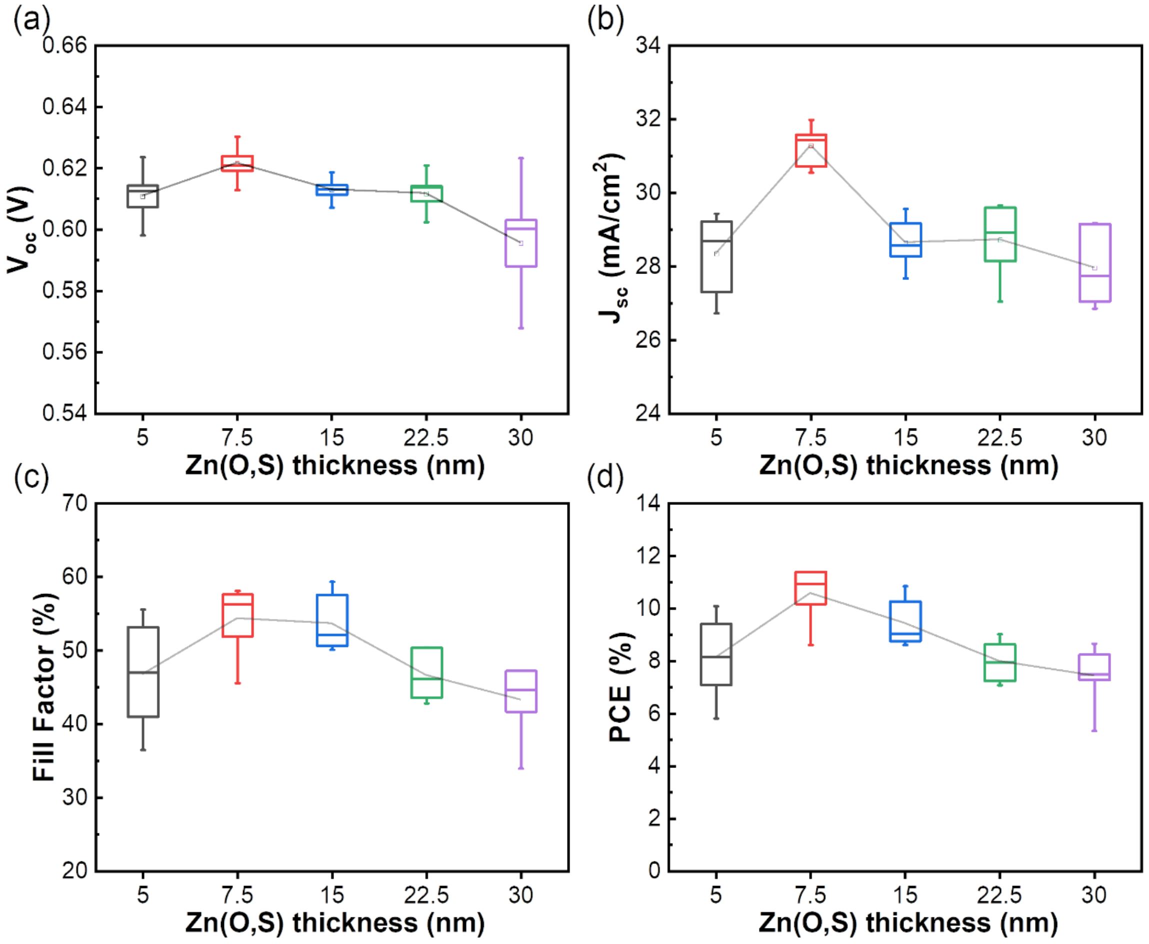

Figure S3. The distribution of PV parameters in STUT CIGSe solar cells with different thicknesses of Zn(O,S) buffer layers (total 30 solar cells). Lower and upper ends of the box indicate 25 and 75% values, whereas upper and lower whiskers located at extreme ends of each distribution represent the highest and lowest values, respectively.

Table S3. Summary of the PV properties of STUT CIGSe solar cells with different ZnO to ZnS cycle ratio of Zn(O,S) buffer layers (total 30 solar cells) showing the average and standard deviation.

Zn(O,S) layer [nm]	V_{oc} [V]	J_{sc} [mA/cm^2]	FF [%]	PCE [%]
5	0.6108±0.008	28.34±1.101	46.71±7.313	8.119±1.586
7.5	0.6215±0.005	31.27±0.5487	54.31±4.811	10.57±1.089
15	0.6129±0.003	28.63±0.6645	53.64±3.859	9.428±0.9114
22.5	0.6116±0.006	28.71±0.9988	46.58±3.472	7.976±0.8166
30	0.5956±0.018	27.95±1.019	43.23±5.092	7.422±1.146

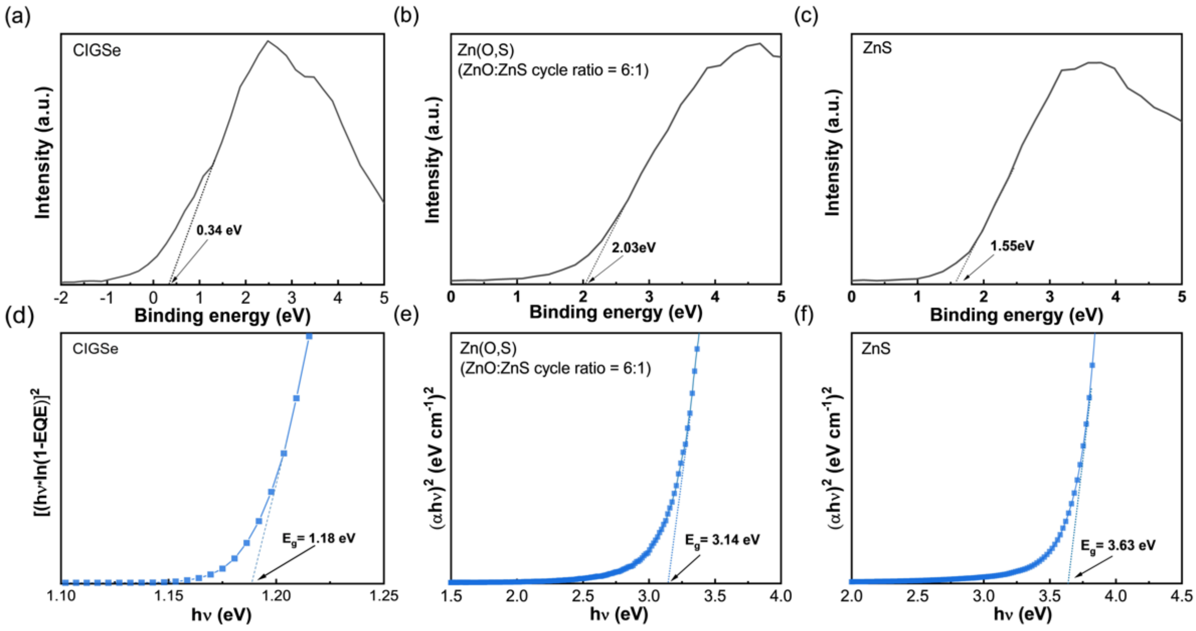

Figure S4. Valence band edge and energy band gap of (a, d) CIGSe, (b, e) Zn(O,S) with ZnO to ZnS cycle ratio of 6:1, and (c, f) ZnS thin films from XPS, EQE, and optical spectra analyses.
